# Gestational saccharin consumption disrupts gut-brain axis glucose homeostasis control in adolescent offspring rats in a sex-dependent manner

**DOI:** 10.1186/s13293-025-00724-5

**Published:** 2025-06-16

**Authors:** Beatriz Pacheco-Sánchez, Sonia Melgar-Locatelli, Raquel López-Merchán, María José Benítez-Marín, Marta Blasco-Alonso, Ernesto González-Mesa, Rubén Tovar, Pablo Rubio, Juan Suárez, Carlos Sanjuan, Fernando Rodríguez de Fonseca, Francisco Alén, Marialuisa de Ceglia, Patricia Rivera

**Affiliations:** 1 Unidad de Gestión Clínica de Salud Mental. Hospital Universitario de Málaga, 29010 Málaga, Spain; 2 Instituto de Investigación Biomédica de Málaga y Plataforma en Nanomedicina-IBIMA Plataforma BIONAND, Málaga, Spain; 3 Departamento de Psicobiología y Metodología de las Ciencias del Comportamiento, Universidad de Málaga, Málaga, Spain; 4 Departamento de Nutrición y Bromatología, Universidad de Granada, Campus Universitario de Cartuja, Granada, Spain; 5 Research Group in Maternal-Fetal Medicine, Epigenetics, Women’s Diseases and Reproductive Health, Instituto de Investigación Biomédica de Málaga y Plataforma en Nanomedicina-IBIMA Plataforma Bionand, 29071 Málaga, Spain; 6 Obstetrics and Gynecology Service, Regional University Hospital of Malaga, 29011 Málaga, Spain; 7 Surgical Specialties, Biochemistry and Immunology Department, Málaga University, 29071 Málaga, Spain; 8 Departamento de Anatomía Humana, Medicina Legal e Historia de la Ciencia, Universidad de Málaga, Bulevar Louis, Pasteur 32, 29071 Málaga, Spain; 9 Euronutra S.L. Calle Johannes Kepler, 3, 29590 Málaga, Spain; 10 Unidad de Gestión Clínica de Neurología, Hospital Regional Universitario de Málaga, 29010 Málaga, Spain; 11 Departamento de Psicobiología, Facultad de Psicología, Universidad Complutense de Madrid, 28040 Madrid, Spain

**Keywords:** Non-nutritive sweeteners, Saccharin, Gestation, Development, Glucagon-like peptide-1, Insulin, Toxicology

## Abstract

**Background:**

Certain events that occur in early life, such as changes in nutrition, can promote structural and functional modifications in brain development, projecting to either short, medium, and/or long terms, resulting in metabolic programming. These effects depend on the timing, intensity, and duration of exposure, and are proposed to be the cause or contribute to chronic adult disorders. Recent studies have proposed that artificial non-nutritive sweeteners (NNS), such as saccharin, can be included as one of these developmental disruptors. Saccharin consumption during pregnancy is strongly discouraged, as it can cross through the placenta and accumulate in the fetus, potentially impacting metabolic control for life. However, the mechanisms underlying the metabolic syndrome induced by maternal NNS consumption during pregnancy are not well understood. Some studies suggest that NNS may affect sweet taste receptors in the adult’s guts, leading to changes in the release of glucagon-like peptide-1 (GLP-1) and insulin. The objective of the study is to investigate whether maternal saccharin consumption during pregnancy affects the gut-brain connection, leading to alterations in insulin/GLP-1 signaling during neurodevelopment until adolescence.

**Methods:**

Pregnant rats were administered 0.1% saccharin in drinking water throughout gestation, and the main components of the insulin/GLP-1 signaling pathway were analyzed in the plasma, small intestine and hypothalamus of the offspring after weaning. Perinatal exposure to saccharin was linked to disrupted glucose homeostasis and insulin sensitivity in both male and female offspring.

**Results:**

We identified sex-dependent mechanisms that affected GLP-1 signaling in the intestine, associated with the expression of taste receptors and glucose transporters. These alterations affected the gut-brain axis and disrupted hypothalamic signaling associated with glucose regulation and food intake, primarily involving the GLP-1, leptin, and insulin signaling pathways.

**Conclusions:**

These results suggest that developmental NNS exposure might contribute to the growing alteration in energy metabolism.

## Highlights

- Perinatal saccharin exposure affects glucose homeostasis in offspring.
- Sex-dependent mechanisms impair GLP-1 signaling in the intestine.
- Changes disrupt gut-brain axis and hypothalamic signaling.
- Findings raise concerns about long-term effects of artificial sweeteners.

## Abstract (plain language summary)

Certain early life experiences, such as dietary changes, can significantly impact brain development, potentially leading to long-term health issues. Recent research indicates that artificial sweeteners, particularly saccharin, may disrupt this developmental process. Pregnant women are advised against consuming saccharin, as it can cross the placenta and accumulate in the fetus, possibly affecting their health throughout life. However, the exact mechanisms by which these sweeteners contribute to health problems in adults remain unclear. Some studies suggest that artificial sweeteners may alter the body’s response to sugar, influencing hormones like GLP-1 and insulin. This study aimed to investigate whether maternal saccharin consumption during pregnancy affects the gut-brain connection, which could alter insulin and GLP-1 signaling during brain development up to adolescence. Pregnant rats were given water containing 0.1% saccharin throughout their pregnancy, and key components of the insulin and GLP-1 pathways were analyzed in the blood, gut, and brain of the offspring after weaning. The findings revealed that exposure to saccharin before birth was associated with issues in sugar and insulin regulation in both male and female offspring. Notably, there were sex-dependent differences in how GLP-1 functioned in the intestines, linked to taste receptors and sugar transport. These alterations impacted the gut-brain connection and disrupted brain signals that regulate sugar levels and food intake, primarily involving GLP-1, leptin, and insulin pathways. Overall, the study suggests that exposure to artificial sweeteners during development may contribute to rising energy management issues.

## Background

Replacing sugary foods and beverages with non-nutritive sweeteners (NNS) is usually a common choice in pregnant women to avoid greater than recommended weight gain. Excessive weight gain along gestation might increase the risk of gestational diabetes, hypertension, preeclampsia and premature birth [1, 2]. Most of these artificial sweeteners are allowed during pregnancy, as long as the acceptable daily intake is not exceeded; however, the consumption of some such as cyclamate, saccharin and raw stevia should be avoided [3].

Controversy exists in the use of NNS to prevent excessive weight gain since observational studies showed a positive correlation between NNS consumption and body weight gain [4, 5]; however, other studies indicate that replacing sugars with NNS induces weight loss, especially in people with excess body weight [6, 7].

Studies in animal models have demonstrated maternal-to-fetal transmission of NNS such as sucralose, acesulfame K and saccharin, suggesting that in utero transmission is likely to occur via the umbilical cord or by NNS filtering through the placenta [8, 9]. In the case of saccharin, it was found in amniotic fluid, fetal bladder and maternal blood in similar quantities [10]. This exposure to sweeteners during the intrauterine period has been described to have a large impact on the programming of offspring’s risk of developing diseases, such as metabolic syndrome, throughout life [3, 8, 11, 12].

However, the mechanisms associated with the metabolic syndrome induced by fetal programming due to maternal consumption of NNS during pregnancy are poorly studied. In this sense, some studies hypothesize an NNS-mediated activation of sweet taste receptors in the intestine, increasing glucose absorption. Additionally, NNS might result in important changes in the offspring’s microbiome [13–15]. Type 1 sweet receptors, on the tongue and in the enteroendocrine cells of the small intestine, detect the sweet taste (T1R1 and T1R3) and are also involved in the enteroendocrine secretion of GLP1. Araujo et al., [13] showed that the activation of TR1 by saccharin increased intracellular Ca2 + and consequently the translocation of the glucose transporter GLUT2, resulting in enhanced glucose absorption capacity in the intestine. The final consequence is the appearance of hyperglycemia in NNS-exposed neonates.

These saccharin-induced alterations in the intestine have consequences in the brain through the gut-brain axis, especially affecting areas sensitive to insulin signaling and integrating peripheral and central signaling regulating food intake, such as the hypothalamus. In this sense, NNS triggers satiety signals in the hypothalamus associated with the release in the gastrointestinal tract and blood of hormones or peptides, such as insulin, ghrelin, peptide YY and the incretins glucagon-like peptides 1 (GLP-1) and 2 (GLP-2) and glucose-dependent insulinotropic peptide (GIP), which in turn target hypothalamic areas that regulate appetite and energy metabolism [16]. Although NNS consumption in adults induced a much smaller reward response than other sweeteners and did not induce changes in the release of the mentioned hormones and peptides [17], perinatal exposure to NNS appears to alter the development of central pathways that are critical for energy regulation.

To further investigate whether gestational exposure to NNS affects the enteroendocrine system of the fetus, in this study we investigated whether saccharin exposure during pregnancy can induce modifications in the gut-brain axis of offspring at weaning age by altering the GLP-1/insulin signaling pathway in the gut and hypothalamus. Studies were performed in offspring of both sexes to unveil potential sex-dependent differences affecting metabolic programming imposed by NNS exposure.

## Methods

### Ethics statement

All experimental procedures with animals were conducted in strict accordance with the guidelines of Royal Decree 1201/2005 of October 21, 2005 (BOE nº252), and in accordance with Directive 86/609/ECC of the European Community (November 24, 1986) in relation to the regulation of animal research. The protocol was approved by the Animal Ethics Committee of the Complutense University of Madrid (PSI-2012–35388; January 2012).

### Animal model

Ten adult female Wistar rats (Charles River Laboratories, Saint-Germain-Nuelles, France) were singly housed in standard cages, with ad libitum access to water and food in a temperature- and humidity-controlled room (22 ± 2 °C and 55 ± 5%, respectively) on a 12-h light/dark cycle. The presence of sperm determined gestational day 0 (GD0). Rats were divided into two groups: saccharin and control. The saccharin group (N = 5) was administered during the entire gestation with 0.1% (weight/volume; g/mL) saccharin (Merck Millipore) diluted in the usual water bottle, and those in the control group (N = 5) drank only water. All mothers drink water during breastfeeding.

At 14 h after birth (PND0), pups were weighed and sexed. The litter size was arranged to comprise 10 pups consisting of 5 males and 5 females where possible. The remaining pups were quickly sacrificed by decapitation. All litters were equally represented in each offspring group.

Half of the pups were used to carry out the glucose and insulin curves the day before sacrifice (N = 7–9), while the remaining pups (N = 6–8) were sacrificed on the day of weaning (PND21) to perform the rest of the analysis.

### Glucose and insulin tolerance test

Half of the pups were used to carry out the glucose and insulin curves the day before sacrifice (N = 7–9). Pups were separated overnight, with free access to water, for 8 h prior to the GTT/ITT tests. For GTT, blood glucose was measured at 0, 30, 60 and 120 min after intraperitoneal glucose administration (20% glucose solution; 10 ml/kg). For ITT, the animals were fasted for 12 h and blood glucose was measured at 0, 15, 30, 45, 60 and 120 min after intraperitoneal administration of insulin (Novo Nordisk) at a low dose (0.5 IU/kg).

Glucose levels were subsequently determined by glucose oxidase method (ACCU-CHEK^®^ Performa, Roche).

### Sample collection

The pups were sacrificed after weaning (PND21). The animals were anaesthetized with pentobarbital (50 mg/kg) and sacrificed by decapitation. Blood samples were collected in tubes containing 6% EDTA and protease inhibitors (Roche cOmplete tablets), and centrifuged (1500 g for 10 min at 4 °C). The plasma was kept at − 80 °C until hormone analysis. Guts and brains were extracted and stored at − 80 °C. The hypothalamus was precisely dissected according to the Bregma coordinates following the Paxinos atlas [18].

### Glucose, insulin and GLP-1 levels

Glucose concentrations were measured with a commercially available glucometer (AccuCheck, Roche, Germany) from the tail vein (basal level) at the moment of sacrifice.

Plasma levels of insulin and GLP-1 were determined by using commercial enzyme-linked immunosorbent assay (ELISA) kits: rat/mouse insulin ELISA kit (EZRMI-13 K, Millipore) and Multispecies GLP-1 ELISA Kit (cat. No. BMS2194, ThermoFisher Scientific).

### RNA isolation and real-time quantitative PCR analysis

Real-time qPCR was performed using specific sets of primer probes from TaqMan Gene Expression Assays (ThermoFisher Scientific, Waltham, MA, USA; Table 1). Selected genes monitored in the intestine included the GLP-1 receptor 1 (*Glpr1*), the taste receptor type 1 member 3 (Sweet receptor or *Tas1R3*), pyy polypeptide (*Pyy*), fatty acid translocase (*CD36*), sodium/glucose cotransporter (*Slc5a1*) and proglucagon (*Gcg*). In the hypothalamus selected gene included also a) feeding regulators such as the agouti-related protein (*Agrp*), Neuropeptide Y (*Npy*), orexin/hypocretin (*Hcrt*) and Leptin receptor (*LepR1*), and b) insulin-related genes including the insulin receptor (*InsR*), insulin receptor substrate 1 and 2 (*Irs1* and *Irs2*), and insulin-induced genes 1 and 2 (*Insig1* and *Insig2*). RNA from the gut and hypothalamus was extracted following the Trizol method according to the manufacturer’s instructions (ThermoFisher Scientific). A melting curve analysis was performed to ensure that only a single product was amplified. After considering several control genes, values obtained from the samples were normalized to Actb levels, which did not vary significantly between groups. Gen expression of the male offspring of control mothers was arbitrarily set as one.

**Table 1:** Primer references for Taqman^®^ Gene Expression Assays

Gene symbol	Assay ID	Amplicon length (bp)
*Actb*	Rn00667869_m1	91
*Insr*	Rn00690703_m1	68
*Irs1*	Rn02132493_s1	147
*Irs2*	Rn01482270_s1	61
*Insig1*	Rn00574380_m1	68
*Insig2*	Rn00710111_m1	89
*Rrad*	Rn00584263_m1	100
*Glpr1*	Rn00562406_m1	87
*Tas1r3*	Rn00590759_g1	82
*Pyy*	Rn01460420_g1	84
*Cd36*	Rn02115479_g1	122
*Slc5a1*	Rn01640634_m1	64
*Gcg*	Rn01485213_m1	65
*Lepr*	Rn01433205_m1	94
*Agrp*	Rn01431703_g1	67
*Pomc*	Rn00595020_m1	92
*Npy*	Rn00561681_m1	63
*Hcrt*	Rn00565995_m1	148

### Western blot analysis

Western blotting was performed as described previously [19]. Briefly, the small intestine and hypothalamus were homogenized in a cold lysis buffer and centrifuged to obtain a supernatant. Protein concentration was measured using the Bradford method, and 30 µg of each sample was separated into polyacrylamide gels. The gels were transferred to nitrocellulose membranes, stained, and blocked before incubation with primary antibodies overnight (Table 2): glucagon-like peptide 1 (GLP-1), glucagon-like peptide 1 receptor (GLP1R), taste receptor type 1 member 3 (Sweet receptor or TAS1R3), fatty acid translocase (CD36), insulin receptor substrate 1 (IRS-1) and its phosphorylated forms at serine 612 (p-IRS1 Ser612) and tyrosine 896 (p-IRS1 Tyr896), phosphoinositide 3-kinases (PI3K) and its phosphorylated form at tyrosine 607 (p-PI3K Tyr607), protein kinase B (AKT) and its phosphorylated form at serine 473 (p-AKT Ser473), and Glycogen synthase kinase-3 beta (GSK3B) and its phosphorylated form at serine 9 (p-GSK3β Ser9). After washing, secondary antibodies were added, followed by visualization of protein bands using a chemiluminescence method. Results were expressed as the ratio of target protein to beta actin. Protein expression of the male offspring of control mothers was arbitrarily set as one.

**Table 2:** Antibodies used for protein expression by Western blotting

Antigen	Manufacturing details	Source	Dilution
b-Actin	Sigma #2535L	Mouse	1/2000
CD36	Abcam #ab252923	Rabbit	1/500
GLP1R	Abcam #ab39072	Rabbit	1/500
TAS1R3	NB100-98792	Rabbit	1/500
GLP1	Abcam #ab23472	Rabbit	1/500
IRS1	Sigma Aldrich #06–248	Rabbit	1/10000
p-IRS1 (Ser612)	Cell Sign #3203	Rabbit	1/1000
p-IRS1 (Tyr896)	Abcam #ab46800	Rabbit	1/500
PI3K	Cell Sign #4257	Rabbit	1/1000
p-PI3K (Tyr607)	Abcam #ab182651	Rabbit	1/1000
AKT	Cell Sign #9272	Rabbit	1/1000
p-AKT (Ser473)	Santa Cruz #sc-16646R	Rabbit	1/1000
GSK3β	Cell Sign #12,456	Rabbit	1/1000
p-GSK3β (Ser9)	Cell Sign #5558	Rabbit	1/1000

### Statistical analysis

Data were expressed as the mean ± standard error of the mean (Standard Error Mean, SEM). Statistical analysis of these data was carried out using a two-way ANOVA (factors: sex and saccharin), using GraphPad Prism 8.0.2 software. Subsequently, multiple comparisons between groups were conducted using different post hoc tests. A simple effects analysis (Fisher’s test) was used when there was no interaction between the factors. In the cases where there was an interaction between sex and saccharin, the Tukey test was applied. A *P*-value less than 0.05 was considered statistically significant.

## Results

### Effect of prenatal saccharin on glucose tolerance and insulin tolerance test

**Fig. 1:**
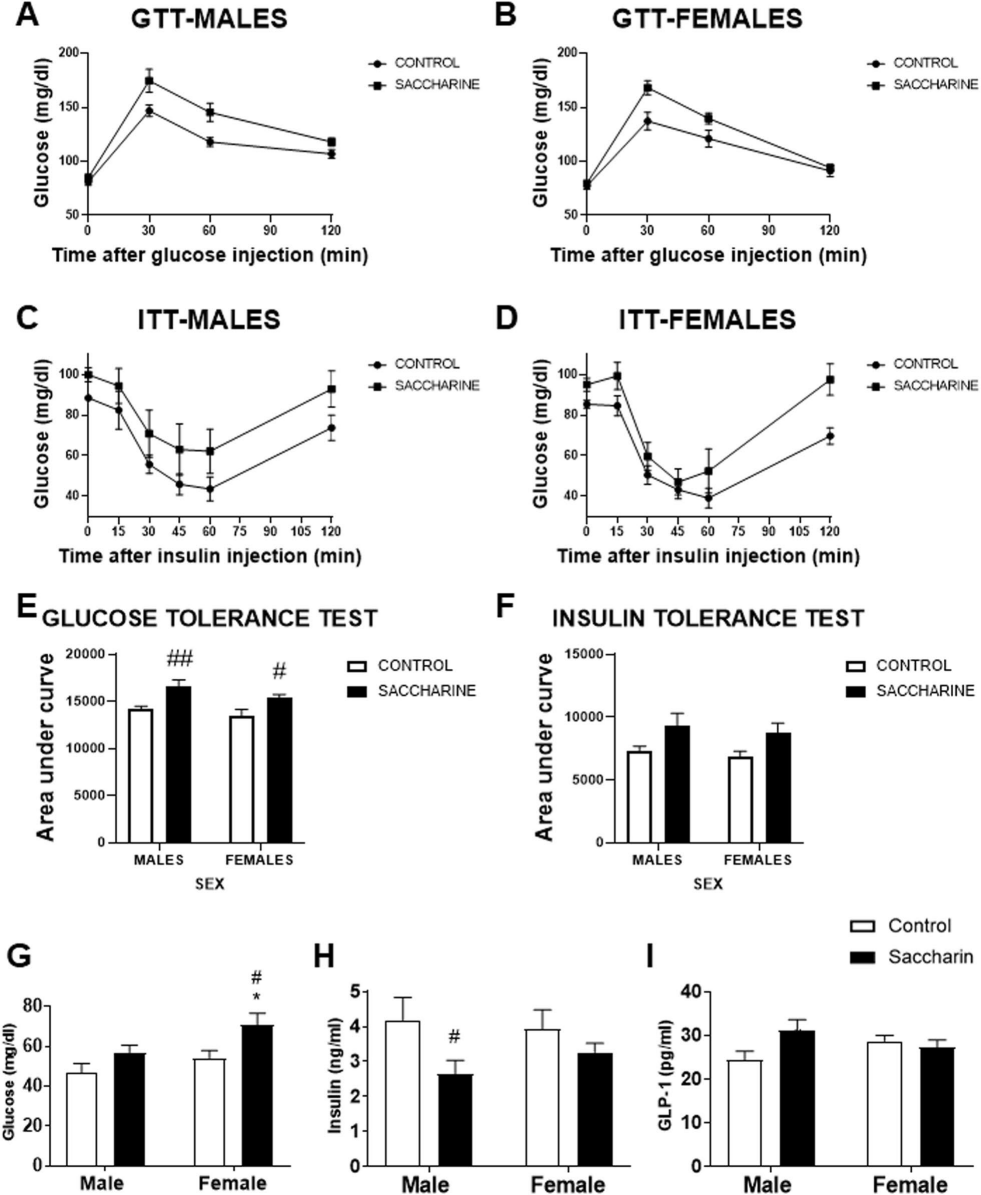
Glucose, insulin and GLP-1 adolescent offspring plasma levels. Glucose tolerance test (GTT; **A**, **B**, **E**) and insulin tolerance test (ITT; **C**, **D**, **F**). Glucose (**G**), insulin (**H**) and GLP-1 (**I**) measurements in blood of male and female adolescent offspring. Data are presented as mean ± SEM and analyzed by two-way ANOVA. (n = 10–33 for each experimental group). Significant post-hoc analysis are shown: *p < 0.05 versus male within the same perinatal conditions; #p < 0.05, ##p < 0.01, ###p < 0.001 versus control within the same sex

Maternal saccharin consumption during gestation had a market impact on glucose tolerance (measured by glucose tolerance test, GTT) and insulin responsiveness (measured by insulin tolerance test, ITT) in offspring at weaning age (Fig. 1d–f). In GTT, Two-way ANOVA evidenced a significant effect for saccharin exposure [F (1,88) = 15.96; p < 0.001] with both male and female offspring showing a significant increase in the area under the curve (AUC) when compared to control offspring (#/##p < 0.05/0.01; Fig. 1e). A similar result was also obtained for ITT, where two-way ANOVA evidenced a significant effect for saccharin exposure [F (1,58) = 6.898; p < 0.05]. Overall, AUC of both male and female saccharin offspring was higher compared to control (Fig. 1f).

### Effect of prenatal saccharin on glucose, insulin and GLP-1 plasma levels of the offspring

Two-way ANOVA showed effects of sex and prenatal saccharin on the glucose levels of adolescent offspring [F (1,89) > 4.125; p < 0.05]; with a general increase in basal glucose in offspring born to mothers who consumed saccharin during gestation, which is significant in females (^*/#^*p* < 0.05; Fig. 1g).

We also observed an effect of prenatal saccharin on the insulin levels of the offspring [F (1,33) = 4.509; p < 0.05]. Post hoc analysis showed an overall decrease in basal insulin levels in offspring born to mothers who consumed saccharin; this decrease was significant in saccharin males compared to control males (^#^*p* < 0.05; Fig. 1h).

An interaction was also found in the adolescent offspring GLP-1 levels [F (1,32) = 5.057; p < 0.05]. No differences between groups were indicated by post hoc test (Fig. 1i).

### Effect of prenatal saccharin on glucose homeostasis gene and protein expression in adolescent offspring small intestine

**Fig. 2:**
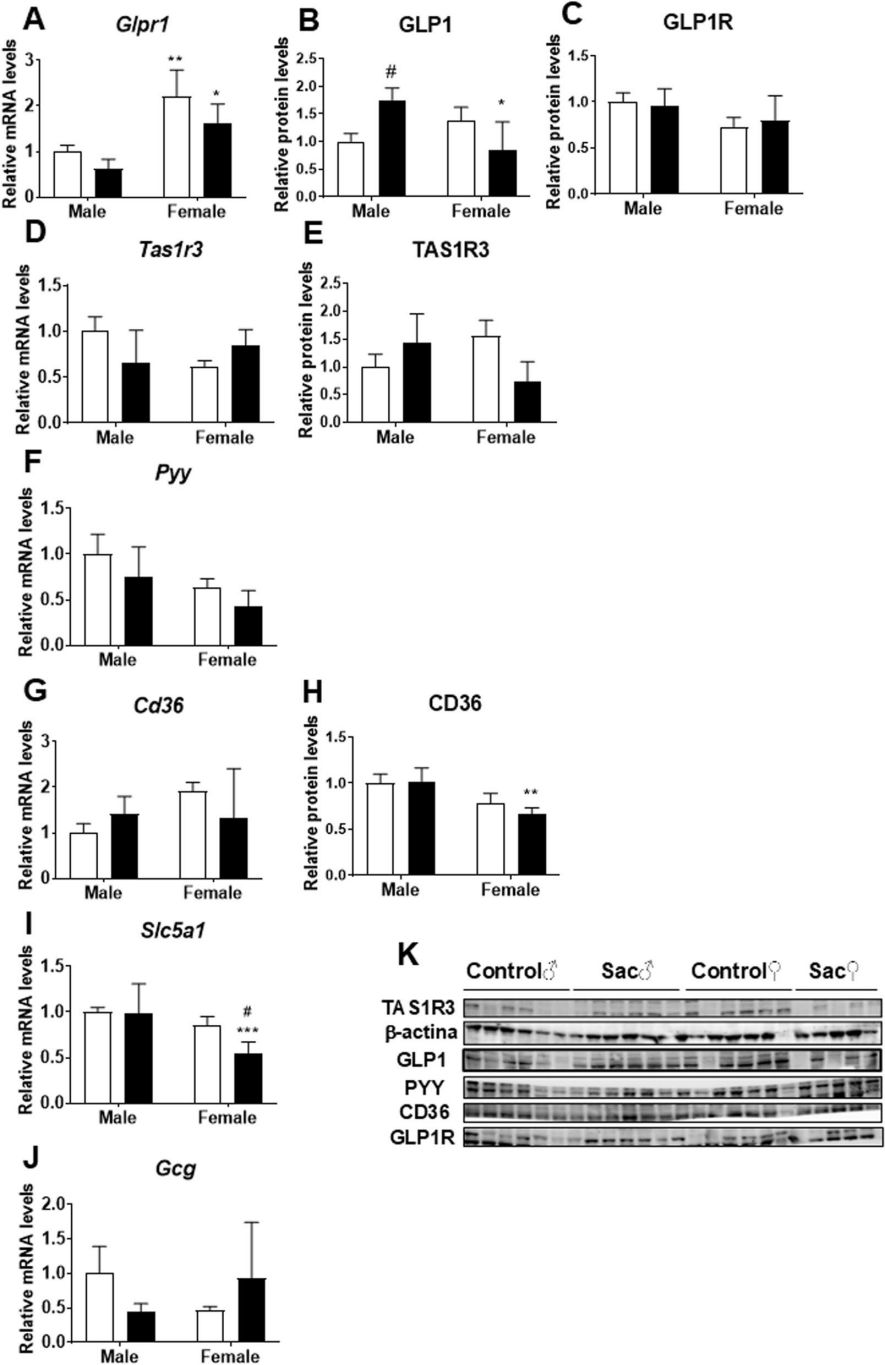
Glucose homeostasis gene and protein expression in adolescent offspring small intestine. Study of gene expression of *Glp1r* (**a**)*, Tas1r3* (**d**)*, Pyy* (**f**)*, Cd36* (**g**)*, Slc5a1* (**i**)*, Gcg* (**j**) by qPCR (n = 9 for each experimental group) and protein expression of GLP1 (**b**), GLPR1 (**c**), TAS1R3 (**e**), CD36 (**h**) by Western blot (n = 6 for each experimental group) in the small intestine of male and female adolescent offspring. (**k**) Representative blots immunostained for each of the proteins tested. Data are presented as mean ± SEM and analyzed by two-way ANOVA. Significant post-hoc analysis is shown: *p < 0.05, ***p < 0.001 versus male within the same perinatal condition; ##p < 0.01 versus control within the same sex

A sex effect on *Glp1r* mRNA levels was observed [F (1,32) = 12.84; p < 0.01]; post hoc analysis showed higher levels of *Glp1r* in the small intestine of females than male groups (^*/**^p < 0.05/0.01; Fig. 2a). An interaction between sex and prenatal saccharin was found on the protein levels of total GLP1 [F (1,20) = 12.20; p < 0.01]. Post hoc analysis showed an overall increase of GLP1 protein level in the gut of male offspring born to mothers who consumed saccharin during gestation (^#/*^p < 0.05; Fig. 2b). Two-way ANOVA also indicated a sex effect on the protein levels of GLP1R [F (1,20) = 4.582; p < 0.05] (Fig. 2c).

Two-way ANOVA also indicated an interaction between sex and saccharin on the mRNA levels of Taste 1 Receptor Member 3 (*Tas1r3*) [F (1,32) = 6.936; p < 0.05] (Fig. 2d), but no significant post-hoc was detected. We also found an interaction between sex and prenatal saccharin on the protein levels of TAS1R3 [F (1,20) = 7.798; p < 0.05] (Fig. 2e).

Sex effect was displayed also on *Pyy* mRNA levels [F (1,32) = 6.613; p < 0.05] (Fig. 2f).

Moreover, Two-way ANOVA showed an interaction between sex and prenatal saccharin on *Cd36* mRNA levels [F (1,32) = 4.327; p < 0.05] and a sex effect on protein levels of CD36 [F (1,20) = 12.65; p < 0.01], with the saccharin female group showing significantly less CD36 levels than male group (^**^p < 0.01; Fig. 2g, h).

A sex effect on *Scl5a1* mRNA levels was also found [F (1,32) = 12.84; p < 0.01], with lower levels of Scl5a1 in the small intestine of female offspring born to mothers who consumed saccharin during gestation compared to control females and saccharin male group (^#/***^p < 0.05/0.001; Fig. 2i).

Finally**,** an interaction between sex and prenatal saccharin was found on the mRNA levels of glucagon (*Gcg*) [F (1,32) > 4.327; p < 0.05], but post-hoc analysis showed no differences between groups (Fig. 2j).

### Effect of prenatal saccharin on adolescent offspring body weight and hypothalamic neuropeptides regulating feeding behavior

**Fig. 3:**
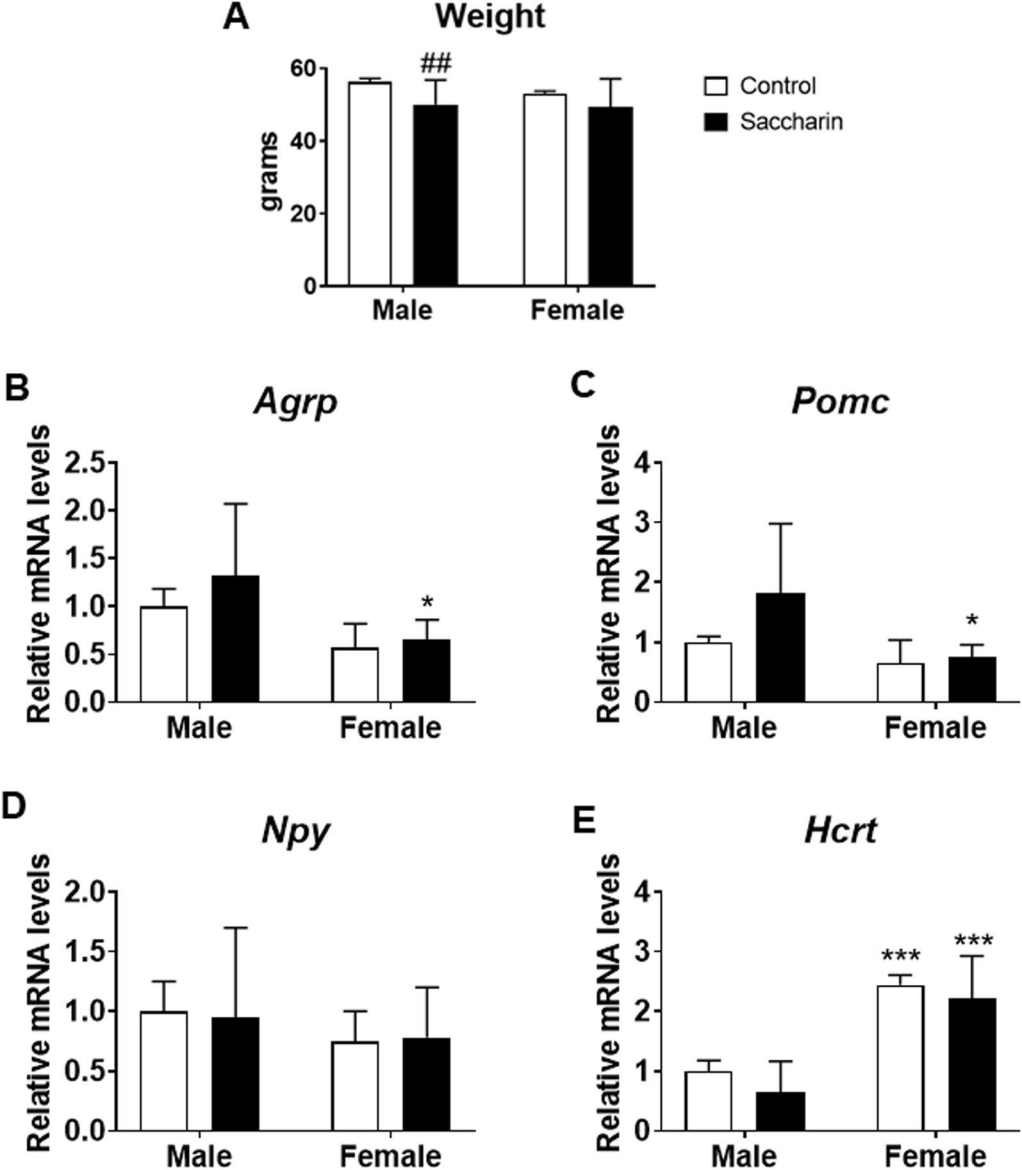
Adolescent offspring body weight and hypothalamic neuropeptides. Body weight of male and female adolescent offspring (**a**) (PND21). Gene expression evaluated by qPCR of *Agrp* (**b**)*, Pomc* (**c**)*, Npy* (**d**)*, Hcrt* (**e**) in the hypothalamus of male and female adolescent offspring. Data are presented as mean ± SEM and analyzed by two-way ANOVA; n = 6 for each experimental group. Significant post-hoc analysis is shown: ##p < 0.01 versus control males; *p < 0.05, ***p < 0.001 versus male within the same perinatal conditions

Two-way ANOVA showed a prenatal saccharin effect on the body weight of adolescent offspring [F (1,58) = 11.99; p < 0.01], with decreased body weight in male offspring from saccharin-mothers compared to control male (^#^*p* < 0.05; Fig. 3a).

Two-way ANOVA also indicated sex effects on the mRNA levels of hypothalamic agouti-related peptide (*Agrp*), proopiomelanocortin (*Pomc*), neuropeptide Y receptor (*Npyr1*) and orexin (*Hcrt*) [F (1,32) > 6.230; p < 0.05]. Post hoc analysis showed lower levels of *Agrp*, *Pomc* and *Npyr1* in the hypothalamus of saccharin female offspring than males (^*^p < 0.05; Fig. 3b–d). We also observed that females showed increased mRNA levels of *Hcrt* compared to males (^***^p < 0.001; Fig. 3e).

### Effect of prenatal saccharin on the hypothalamic expression of genes and proteins related to insulin signaling and metabolism

**Fig. 4:**
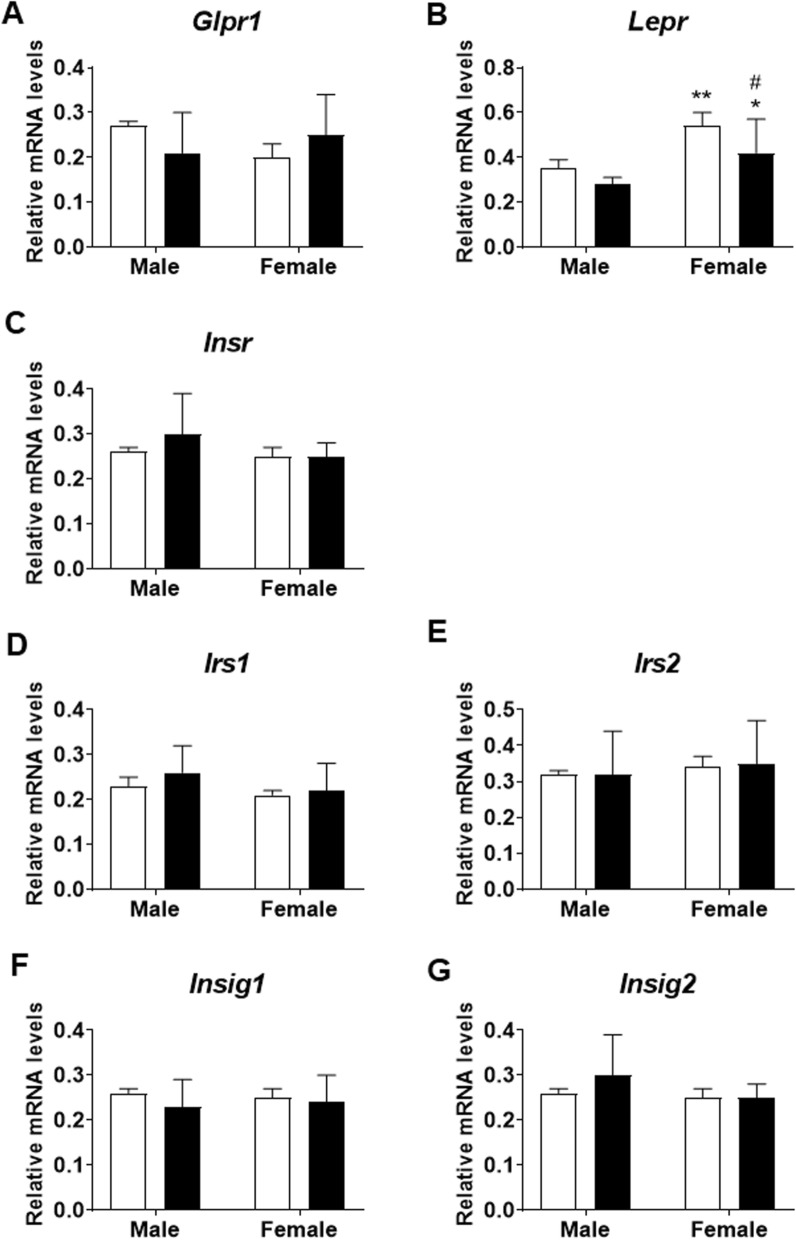
Gene expression on adolescent offspring hypothalamic glucose-related circuits. Gene expression of *Glpr1* (**a**)*, Lepr* (**b**)*, Insr* (**c**)*, Irs1* (**d**)*, Irs2* (**e**)*, Insig1* (**f**)*, Insig2* (**g**) was evaluated by qPCR in the hypothalamus of male and female adolescent offspring. Data are presented as mean ± SEM and analyzed by two-way ANOVA; n = 9 for each experimental group. Significant post-hoc analysis is shown: *p < 0.05, **p < 0.01 versus male within the same perinatal conditions; #p < 0.05 versus control within the same sex

Two-way ANOVA indicated an interaction between sex and prenatal saccharin on *Glp1r* mRNA levels in the hypothalamus of adolescent offspring [F (1,32) = 4.321; p < 0.05]. No differences between groups were indicated by post hoc test (Fig. 4a).

We also observed effects of sex and prenatal saccharin on *Lepr* mRNA levels [F (1,32) > 4.628; p < 0.05]. Post hoc analysis showed higher *Lepr* levels in control and saccharin females than respective male groups (^*/**^p < 0.05/0.01; Fig. 4b), but lower mRNA levels of this receptor in the hypothalamus of saccharin females than control female group (^#^*p* < 0.05; Fig. 4b).

No interaction or effects of sex and prenatal saccharin on *Insr*, *Irs1*, *Irs2*, *Insig1*, and *Insig2* RNAm levels in the hypothalamus of adolescent offspring were found (Fig. 4c, g).

**Fig. 5:**
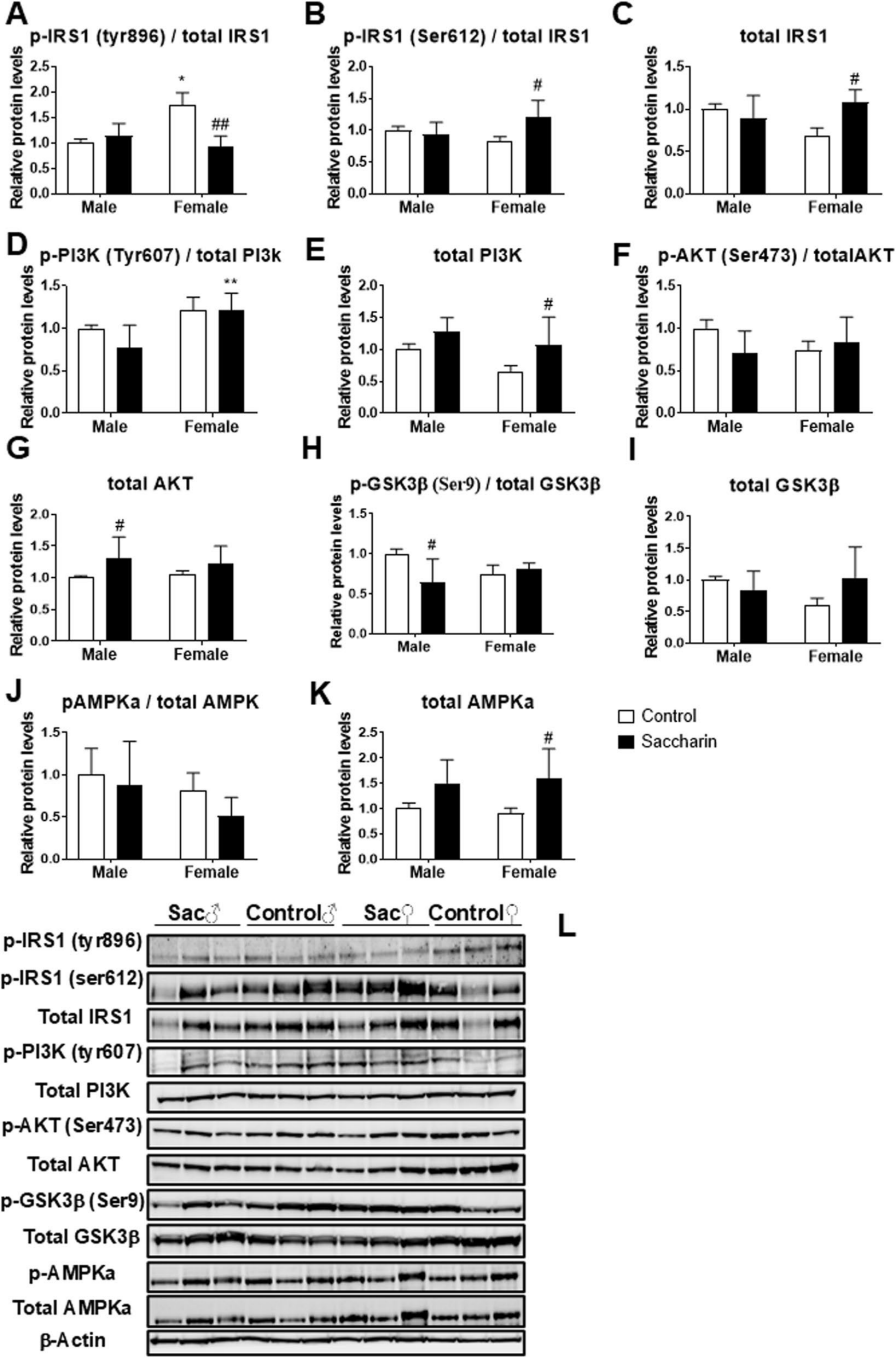
Protein expression in adolescent offspring hypothalamic insulin-related circuits. Protein expression of IRS-1 (phosphorylated in tyr896, (**a**); phosphorylated in ser612, (**b**); total form, (**c**); phosphorylated (**d**) and total PI3K (**e**); phosphorylated (**f**) and total AKT (**g**); phosphorylated (**h**) and total GSK3β (**i**); phosphorylated (**j**) and total AMPKα (**k**) was evaluated by western blot in the hypothalamus of male and female adolescent offspring. (**l**) Representative blots immunostained for each of the proteins tested. Data are presented as mean ± SEM and analyzed by two-way ANOVA; n = 6 for each experimental group. Significant post-hoc analysis is shown: *p < 0.05, **p < 0.01 versus male within the same perinatal conditions; #p < 0.05, ##p < 0.01 versus control within the same sex

Two-way ANOVA indicated a prenatal saccharin effect [F (1,20) = 5.315; p < 0.05] and an interaction between sex and saccharin [F (1,20) = 10.59; p < 0.01] on the protein levels of the phosphorylated form of IRS1 at Tyr896 (p-IRS1). Tukey post hoc analysis showed decreased p-IRS1 in the hypothalamus of female offspring from mothers who consumed saccharin during gestation compared to control females (^##^*p* < 0.01; Fig. 5a).

An interaction between both factors was also found on the protein levels of p-IRS1 (Ser612) and total IRS1 [F (1,20) > 7.105; p < 0.05], with the saccharin female group showing significantly more p-IRS1 (Ser612) and total IRS1 than control group (^#^*p* < 0.05; Fig. 5b, c).

A sex effect on the protein levels of p-PI3K (Tyr607) was also found [F (1,20) = 10.54; p < 0.01], with female saccharin group showing higher levels of p-PI3K than males (^**^p < 0.01; Fig. 5d).

Main effects of sex [F (1,20) = 5.335; p < 0.05] and prenatal saccharin [F (1,20) = 8.397; p < 0.01] on the protein levels of total PI3K were found. Post hoc analysis showed higher levels of total PI3K in the hypothalamus of female offspring from mothers who consumed saccharin during gestation compared to control females (^#^*p* < 0.05; Fig. 5e).

A saccharin effect on the protein levels of total AKT, but not of p-AKT (Ser473), was observed [F (1,20) = 6.293; p < 0.05], with an overall increase in the protein levels of total AKT in male offspring born to mothers who consumed saccharin during gestation compared to control male (^#^*p* < 0.05; Fig. 5f, g).

Interactions between sex and prenatal saccharin were found on p-GSK3β (Ser9) and total GSK3β [F (1,20) > 5.049; p < 0.05]. Post hoc analysis showed lower levels of p-GSK3β in the hypothalamus of male offspring born to mothers who consumed saccharin during gestation compared to control male (^#^*p* < 0.05; Fig. 5h, i).

Two-way ANOVA also showed a saccharin effect [F (1,20) = 12.02; p < 0.05] on the protein levels of total AMPKα, but not of p-AMPKα, with the saccharin female group showing significantly more total AMPKα than control group (^#^*p* < 0.05; Fig. 5j).

## Discussion

Saccharin is a type of NNS widely used in human nutrition, even by pregnant women, although its consequences on offspring are not fully clarified. In this study, we demonstrated that maternal saccharin consumption during pregnancy elevated serum glucose levels, lowered insulin levels, and altered GLP-1 levels in offspring at weaning age, with these effects being modulated by sex. The disruptions in glucose homeostasis were linked to changes in the expression of genes and proteins related to taste receptors and glucose transporters in the offspring’s small intestine. In addition, these changes were associated with important protein alterations in the hypothalamus, the main brain region involved in the control of metabolism.

First, prenatal saccharin consumption resulted in disruption of glucose homeostasis, leading to glucose intolerance at weaning, as reflected on the increased time spent on hyperglycaemia in the GTT test, as in the reduced efficacy of insulin observed in the ITT test. Considering basal levels, saccharine exposure induced an increase in glucose and a decrease in insulin in adolescent offspring, more evident in females and males respectively. Consistently, prenatal exposure to other sweeteners also induced insulin resistance in offspring in a sex-dependent manner; thus Collison et al. 2012 demonstrated that exposure of mice to aspartame in utero and throughout life increased fasting glucose levels. Male offspring exposed to aspartame experienced the highest elevations of fasting blood glucose and decreased insulin sensitivity [20]. In another study, using aspartame in the prenatal period, they demonstrated that male rat offspring exposed to aspartame during gestation had hyperglycemia [21]. Early postnatal exposure to NNS (acesulfame potassium, saccharin, or stevia) significantly impaired glucose uptake in offspring rats; which showed rapid increases in blood glucose levels after intragastric glucose infusion, probably due to alterations in glucose transporters in the proximal gut. Overall, regular ingestion of artificial low-calorie sweeteners during early life led to changes in sugar-related behaviors and glucoregulation in adulthood, influenced by modifications in sweet taste receptors and glucose transport systems [22]. Thus, it seems clear that the use of sweeteners during pregnancy induces hyperglycemia and insulin resistance in offspring; and that the degree of alteration and the most affected sex depends on the dose and time of consumption. The similarity of these findings in the early postnatal age with the results of the present study, focused on the prenatal stage, indicate that early NNS consumption has long-lasting implications for glucoregulation.

The alterations in blood glucose metabolism observed in the offspring were coupled with significant alterations in the expression of glucoregulation-related genes and proteins in the small intestine, which were also sex-dependent.

Saccharin consumption during pregnancy affected the gene and protein expression of the sweet taste receptor Tas1r3. Interestingly, Tas1r3 gene and protein expression in the intestine had opposite directions: we hypothesise that gene expression adopts a compensatory role to the effect induced by prenatal saccharin on the protein expression of this receptor in the small intestine of the offspring. Moreover, consistent with the explanations of Kearns and Reynolds 2024 [23], TAS1R3 protein expression showed the same profile as GLP-1 levels in the plasma and small intestine of offspring at weaning age. Consequently, in male offspring from mothers that consumed saccharin, increased TAS1R3 expression resulted in increased GLP-1 levels in blood and small intestine, whereas the opposite effect was observed in female offspring. This could be justified by the fact that the stimulation of sweet taste receptors by NNS in the small intestine enhances the absorption of glucose and triggers the release of incretin hormones, such as glucagon-like peptide, which play a crucial role in stimulating insulin release from the pancreas [23, 24]. Interestingly, only female offspring of prenatal saccharin showed a significant decrease in Slc5a1 expression, a protein controlling glucose absorption in the intestine. This variation could cause glucose malabsorption [25, 26] and be responsible for the significant increase in blood glucose observed in these animals. Moreover, Slc5a1 controls GLP-1 production; meaning that a decrease in its activity leads to lower GLP-1 production in the intestine as observed in female offspring of saccharin-consuming mothers [27]. The expression of the proglucagon gene, the precursor of GLP-1 but also of glucagon and other peptides, seemed not to be affected profoundly by saccharine, with a minor tendency in the case of males exposed to saccharine. It is possible then that the alteration of GLP-1 production is related to changes in the alternative processing of the proglucagon transcript, a fact that needs to be demonstrated.

A similar trend was observed for CD-36, whose protein expression was significantly decreased in prenatal saccharin female offspring only: GLP-1 is a known regulator of CD-36 expression; and it has been demonstrated that its analogues (as liraglutide), stimulate CD-36 expression [28]; thus meaning that reduced expression of intestinal GLP-1 can be associated with reduced expression of intestinal CD-36 as observed in our experiment. Dysregulations in intestinal signaling related to GLP-1, Slc5a1 and CD-36 can be related with changes in insulin sensitivity [28–30]. On one hand, this evidence justifies the results observed on GTT and ITT in the female offspring of saccharine-exposed rats. On the other hand, the fact that also male offspring of saccharine-exposed rats display increased blood glucose, decreased insulin and altered GTT and ITT suggests the existence of different mechanisms of action, which are sex-dependent. In fact, differently from females, male offspring of saccharine-exposed rats presented an increase in GLP-1 in blood and intestine. Generally, GLP-1 supports glucose metabolism by promoting insulin sensitivity, however, chronically high GLP-1 levels can contribute to insulin resistance through GLP-1 receptor desensitisation and cause reduced blood insulin secretion and increased blood glucose, as observed in these animals [31, 32]. This hypothesis is sustained by the fact that a trend of decreased gene and protein expression of the intestinal GLP-1 receptor was observed in saccharine male offspring. Enteroendocrine L cells secrete GLP-1 in response to sugars or artificial sweeteners into the intestinal lumen [33]; from splicing of pro-glucagon gene (Gcg). Increased intestine GLP-1 levels in male offspring provoked a decrease in genetic expression of its precursor Gcg in the same animals, due to physiological feedback: downregulation of proglucagon synthesis to maintain balance and avoid excessive GLP-1 levels [34].

The disrupted signals at blood and intestinal levels in perinatal saccharin offspring reach the hypothalamus, the most important region controlling energy expenditure and food intake [35, 36] via the gut-brain axis. NNS can influence gut-brain signaling by releasing intestinal peptides and activating vagal afferent pathways, and this signaling may play an important role in glucose homeostasis [24, 37]. Among the intestinal peptides influenced by NNS we can find GLP-1 and PYY; which are normally coexpressed. Interestingly, in saccharin offspring both peptides showed gene or protein alterations, suggesting disruptions in the gut-brain axis of these animals. In particular, mRNA levels of PYY were reduced in both male and female saccharin offspring, suggesting alterations in insulin secretion, due to PYY role in pancreas and hypothalamus [38]. Taken together, these alterations suggest that perinatal exposure to saccharin may affect the gut-brain axis.

Consistently with previous studies demonstrating that NNS fail to satisfy the hedonic aspects of food consumption and do not affect hypothalamic appetite system because they do not activate anorexigenic neuropeptides [39, 40], we demonstrated that perinatal exposure to saccharin does not impact the expression of genes encoding orexigenic and anorexigenic hormones (such as Agrp, Pomc, Npy, Hcrt) at weaning; even though significant changes were noticed between male and female animals regardless of saccharin gestational exposure.

On the other hand, some changes in the expression of hypothalamic receptors of peripherally-produced hormones were observed. Altered production of GLP-1 in the intestine resulted in disrupted expression of GLP-1 receptor in the hypothalamus of both saccharin-exposed groups. Likewise, a decrease in the expression of leptin receptors was observed in both sexes from saccharin-consuming mothers, being significant in females. Evidence demonstrated that GLP-1 and leptin receptors interact at the hypothalamic level to regulate glycemia, with mechanisms yet to be fully discovered [41, 42]: this could be another factor influencing the disrupted ITT and GTT observed in perinatal saccharin offspring. On the contrary, no significant changes were observed in the gene expression of *Insr* or genes related to insulin pathway (*Irs, Insig*) even though significant alterations in blood insulin levels of saccharin offspring were observed. However, significant variations in the expression of proteins related to the insulin pathway were noticed.

GLP-1, insulin and leptin share several common signaling pathways in the hypothalamus, particularly involving kinases and intracellular cascades that regulate energy balance, glucose homeostasis, and appetite. Interestingly, only female offspring of saccharin-consuming mothers show disruptions in IRS-1 signaling at protein level with decreased phosphorylation at tyr896, increased phosphorylation at ser612 and increased total IRS-1. Such changes have been associated with insulin resistance and variations in glucose homeostasis in various models, including one of preclinical intrauterine growth restriction [43, 44]. Evidence demonstrated a mechanism linking the leptin receptor to IRS-PI3K signaling [45]: in female offspring, altered IRS-1 and leptin receptor expression leads to a significant increase in PI3k phosphorylation and total PI3k expression. PI3K activation in the hypothalamus plays a key role in suppressing appetite, triggering satiety and reducing food intake [46]. On the other hand, male offspring of saccharin-consuming mothers showed no significant variations in IRS-1 or PI3K, but presented a significant decrease in phosphorylation of GSK3b, reducing its activation and consequently causing alterations in AKT pathway (whose total protein expression was significantly increased) [47]. Disrupted GSK3b activity can negatively impact glucose metabolism by interfering with insulin signaling, leading to impaired glucose regulation, as observed in the ITT and GTT of perinatal saccharine offspring and also compromise hypothalamic signaling pathways that regulate hunger and satiety [48, 49]. Importantly, PI3K and AKT are important downstream regulators of the GLP-1 pathway [50] and are involved in the suppression of food intake. Moreover, studies with the GLP-1 agonist Exenatide suggested the existence of an insulin-IRS-1-PI3K/AKT-dependent pathway that mediates the food intake-suppressing effects of the GLP-1 receptor in the arcuate nucleus [51]. Altered GLP-1 signaling observed in the intestine and hypothalamus of saccharine offspring drives changes in the expression of these proteins, towards an anorexigenic signal. In addition, offspring of both sexes exposed to perinatal saccharin showed an increase in AMPKα protein expression (being significant in females). Normally, insulin inhibits AMPKα activity in several hypothalamic regions, such as the PVH, ARC, and LHA, which contribute to its anorexigenic effect. Similarly, GLP-1 also exerts an anorectic effect by inhibiting AMPK activation in the hypothalamus [52]. However, under the condition of our experiment, perinatal saccharine disrupts both GLP-1 and insulin signaling, resulting in increased activation of AMPKα and anorexigenic activity.

As confirmation of the anorexigenic action exerted by these significant variations in saccharin adolescent offspring, we observed a significant reduction in the body weight of the animals that were perinatally exposed to saccharin when compared to their control littermates.

## Conclusions

In conclusion, our research evidenced that perinatal exposure to saccharin was associated with impaired glucose homeostasis and insulin sensitivity in both male and female offspring. We could evidence the existence of sex-dependent mechanisms impairing GLP-1 signaling in the intestine, depending mainly on the expression of taste receptors and glucose transporters. These changes provoked variations in the gut-brain axis and disruptions in hypothalamic signaling related to glucose homeostasis and regulation of food intake, involving mainly GLP-1, leptin and insulin signaling pathways.

These findings raise concerns about the long-term effects of artificial sweeteners on metabolic health, particularly during critical developmental windows. Future research should focus on understanding the lasting impact of perinatal NNS exposure, including its potential role in the development of metabolic disorders in adulthood. Additionally, investigating the translational relevance of these findings to human populations could provide critical insights into the safety of artificial sweeteners during pregnancy.

## Data Availability

No datasets were generated or analysed during the current study.
